# HMGB1 Is Involved in the Protective Effect of the PPAR**α** Agonist Fenofibrate against Cardiac Hypertrophy

**DOI:** 10.1155/2014/541394

**Published:** 2014-01-09

**Authors:** Zhankui Jia, Rui Xue, Gangqiong Liu, Ling Li, Jinjian Yang, Guofu Pi, Shengli Ma, Quancheng Kan

**Affiliations:** ^1^Department of Urology, The First Affiliated Hospital of Zhengzhou University, No. 1 Jian She Dong Avenue, Zhengzhou 450002, China; ^2^Department of Cardiology, The First Affiliated Hospital of Zhengzhou University, No. 1 Jian She Dong Avenue, Zhengzhou 450002, China; ^3^Department of Orthopedics, The First Affiliated Hospital of Zhengzhou University, No. 1 Jian She Dong Avenue, Zhengzhou 450002, China; ^4^Department of Pharmacology, The First Affiliated Hospital of Zhengzhou University, No. 1 Jian She Dong Avenue, Zhengzhou 450002, China

## Abstract

High mobility group box 1 (HMGB1) is a ubiquitous nuclear DNA-binding protein whose function is dependent on its cellular location. Extracellular HMGB1 is regarded as a delayed mediator of proinflammatory cytokines for initiating and amplifying inflammatory responses, whereas nuclear HMGB1 has been found to prevent cardiac hypertrophy and heart failure. Because fenofibrate, a peroxisome proliferator-activated receptor **α** (PPAR**α**) agonist, has shown both protective effects against cardiac hypertrophy and inhibitory effects against inflammation, the potential modulation of HMGB1 expression and secretion by fenofibrate is of great interest. We herein provide evidence that fenofibrate modulates basal and LPS-stimulated HMGB1 expression and localization in addition to secretion of HMGB1 in cardiomyocytes. In addition, administration of fenofibrate to mice prevented the development of cardiac hypertrophy induced by thoracic transverse aortic constriction (TAC) while increasing levels of nuclear HMGB1. Altogether, these data suggest that fenofibrate may inhibit the development of cardiac hypertrophy by regulating HMGB1 expression, which provides a new potential strategy to treat cardiac hypertrophy.

## 1. Introduction 

High mobility group box 1 (HMGB1), a nuclear DNA-binding protein, is expressed in diverse cell types, including cardiomyocytes [1, 2]. HMGB1 exhibits diverse functions according to its cellular location. In the extracellular compartment, it plays an important role in inflammatory responses when actively secreted from stressed cells [3]. However, in addition to its extracellular functions, intracellular HMGB1 participates in a number of fundamental cellular processes such as transcription, replication, and DNA repair [4, 5]. HMGB1 has been demonstrated to play a pivotal role in cardiovascular disease in different studies [6]. Additionally, maintenance of stable nuclear HMGB1 levels has emerged as a potential treatment for cardiac hypertrophy because HMGB1 overexpression in the nucleus can prevent hypertrophy and heart failure by inhibiting DNA damage [2].

Peroxisome proliferator-activated receptors (PPARs) are ligand-dependent transcription factors belonging to the nuclear receptor superfamily [7]. There are three known PPAR isoforms, *α*, *β*/*δ*, and *γ*, which exhibit tissue-specific distribution and legend-specific effects [8, 9]. In particular, PPAR*α* is abundant in metabolically active tissues, including the liver, brown fat, kidney, skeletal muscle, and heart [10]. In addition to their metabolic roles in the heart, PPAR*α* and its agonist fenofibrate have received great attention because of their effects related to cardiac inflammation and hypertrophy [11, 12]. In cardiomyocytes, coadministration with fenofibrate inhibits the hypertrophic response induced by endothelin-1 by reducing cardiomyocyte surface area and decreasing protein synthesis [13]. Fenofibrate also reduces the Ang-II induced expression of TGF-*β*1, collagen deposition, and macrophage infiltration during the process of myocardial inflammation [14]. Considering that HMGB1 is highly expressed in cardiomyocytes and promotes both cardiac inflammation and cardiac hypertrophy, we hypothesize that HMGB1 is possibly regulated by fenofibrate. Therefore, we investigated whether activation of PPAR*α* with fenofibrate, a PPAR*α*-specific agonist, modulates HMGB1 expression and localization in cardiomyocytes. Furthermore, we provide insights into a possible mechanism of how PPAR*α* suppresses the progression of cardiac hypertrophy, with HMGB1 involvement. This study reveals a novel role of fenofibrate in modulating HMGB1 expression and provides a new potential mechanism for fenofibrate in treating cardiac hypertrophy.

## 2. Materials and Methods

### 2.1. Reagents

Fenofibrate (catalog: F6020) and LPS (catalog: L3755) were purchased from Sigma-Aldrich (St. Louis, MO). All of the cell culture media and supplements were from Sigma. Antibodies against HMGB1 (catalog: 3935) were from Cell Signaling Technology (Danvers, MA). The anti-PPAR*α* antibody (catalog: ab8934) and anti-histone H3 antibody (catalog: ab1791) were obtained from Abcam (Hong Kong). The anti-actin antibody (catalog: A2668) was obtained from Sigma. Primers for quantitative real-time reverse transcriptase-PCR were designed using GenBank sequences.

### 2.2. Cell Culture

Primary cultures of neonatal rat cardiomyocytes were performed as described previously [15, 16]. In brief, hearts were collected from 1- to 2-day-old neonatal rats promptly after euthanasia by decapitation. The ventricular tissue parts were subjected to multiple rounds of enzymatic digestion with collagenase II (Worthington). The digested samples were then centrifuged at 800 g for 5 min at 4°C and the cells were collected. Nonmyocytes were removed by two rounds of preplating on culture dishes. The enriched cardiomyocytes were cultured in 4 mL of growth medium (Dulbecco's modified Eagle medium supplemented with 10% fetal bovine serum and 10 *μ*M cytosine 1-*β*-d-arabinofuranoside). Addition of cytosine 1-*β*-d-arabinofuranoside inhibits the growth of contaminating nonmyocytes. More than 90% of the cells were cardiomyocytes (positive for *α*-actinin). Cardiomyocytes were washed with PBS the next day to remove unbound cells and were used for experiments at 3 d after the cells stabilized.

### 2.3. Hypertrophic Myocardium Model

Cardiac hypertrophy was induced in mice by thoracic transverse aortic constriction (TAC), a well-known surgical technique that can lead to pressure overload. The TAC model has been previously used to examine the role of HMGB1 [2, 17]. Healthy 10-week-old male mice weighing 24–26 g were randomly divided into the model group and sham group. Thoracic TAC was performed as described by Hu et al. [18]. Briefly, mice were anesthetized by intraperitoneal injection with a mixture of ketamine (80 mg/kg) and xylazine (8 mg/kg), and the aortic arch was exposed under the thymus, without thoracotomy, in spontaneously breathing mice. A 5–0 silk thread was passed under the aorta between the origin of the right innominate and left common carotid arteries and snared around the aorta, and a bent 27-gauge needle was placed alongside the aortic arch. After ligation, the needle was quickly removed, the skin was closed, and the mice were allowed to recover under infrared light until they were fully awake. The sham operation was identical, except that the thread was not ligated.

### 2.4. Echocardiography

Echocardiography was used to determine cardiac parameters in live mice, including interventricular wall thickness (IVS), left-ventricular end-diastolic dimension (LVEDD), and left-ventricular end-systolic dimension (LVESD). Echocardiography was performed on mice sedated with isoflurane vaporized in oxygen. A Vevo 770 high-resolution ECHO system equipped with a 35 MHz transducer was used to obtain images. All of the mice underwent echocardiography at 4 weeks after the TAC or sham surgery.

### 2.5. RNA Extraction and Real-Time PCR

Total RNA from cardiomyocytes and hearts was isolated using Trizol reagent (Life Technologies). Total RNA was reverse-transcribed to cDNA using the TaqMan Reverse Transcription Reagent Kit (Applied Biosystems) according to the manufacturer's protocol. Real-time PCR was performed using an iCycler device with the SYBR Green I probe (Bio-Rad, Hercules, CA). Each sample was analyzed in triplicate and mRNA levels were normalized against the level of GAPDH mRNA. Rat HMGB1 and PPAR*α* mRNA levels, in addition to mouse ANP, BNP, and *β*-MHC mRNA levels, were determined by quantitative real-time RT-PCR. The primer sequences are in Table 1. PCR products were validated by electrophoresis on 2% agarose.

### 2.6. Western Blot Analysis

Cytoplasmic and nuclear proteins of neonatal rat and adult mouse ventricles were extracted using nuclear and cytoplasmic extraction reagents (Pierce, Rockford, IL) according to the manufacturer's instructions. The protein concentration of the myocardial samples was determined using a protein concentration assay (Bio-Rad Laboratories, Hercules, CA). The extracted proteins were boiled in protein buffer for 5 min, separated by 12% SDS-PAGE, and transferred to a nitrocellulose membrane. The membrane was incubated at room temperature for 1 h in TBS-T (Tris-buffered saline containing 0.1% Tween 20) containing 5% skimmed milk to block nonspecific binding sites. The membrane was then incubated overnight at 4°C with the primary antibodies. The membrane was washed for 5 min with TBS-T buffer three times and then incubated with a horseradish peroxide-conjugated secondary antibody at room temperature for 1 h. Finally, the membrane was developed using ECL reagent (Vigorous Biotechnology, Beijing, China) and exposed to Kodak XBT-1 film.

### 2.7. ELISA Analysis for HMGB1 in Medium

ELISA analysis was performed as described previously [19]. The HMGB1 level in cardiomyocyte supernatants was measured using a commercially available ELISA Kit from Shino-Test Corporation (Tokyo, Japan) according to the manufacturer's instructions. Absorbance was measured at 450 nm on an ELISA reader (Bio-Rad Laboratories).

### 2.8. Statistical Analysis

SPSS software was used to analyze the data. All of the real-time PCR and ELISA data are presented as the mean ± standard error of the mean (SEM). All of the experiments were repeated a minimum of three times. Differences between groups were evaluated using a one-way ANOVA analysis of variance with post hoc Bonferroni tests. *P* < 0.05 was considered statistically significant.

## 3. Results

### 3.1. Fenofibrate Inhibits the Basal and LPS-Induced Expression of HMGB1 in Cardiomyocytes

**Figure 1:**
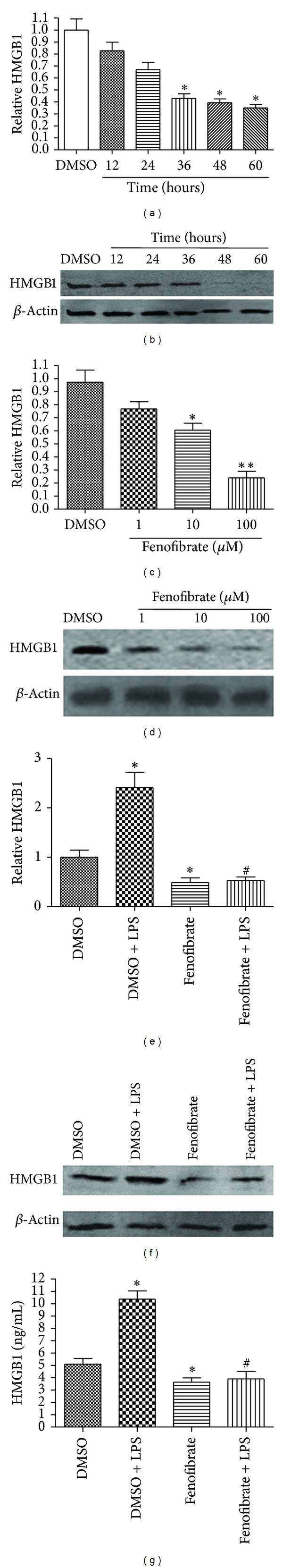
Fenofibrate inhibits the basal and LPS-induced expression of HMGB1. (a) and (b) Fenofibrate induces time-dependent inhibition of the expression of HMGB1 in primary cardiomyocytes at the mRNA level (a) and protein level (b). Cells were treated with 10 *μ*M fenofibrate for 12, 24, 36, 48, and 60 h before being used for experiments. (c) and (d) Fenofibrate-induced concentration-dependent inhibition of the expression of HMGB1 in primary cardiomyocytes at the mRNA level (c) and protein level (d). Cells were treated with 1, 10, and 100 *μ*M fenofibrate for 36 h before experiments. (e) and (f) Fenofibrate inhibited basal and LPS-induced HMGB1 expression in cardiomyocytes as determined by real-time PCR (e) and western blot (f). Cells were treated with the DMSO vehicle or the same dose of fenofibrate at a final concentration of 10 *μ*M for 36 h; LPS (50 ng/mL) was then added and incubated for 10 h. (g) Fenofibrate inhibits basal and LPS-induced secretion of HMGB1 in cardiomyocytes; similar treatments were performed as described for (e) and (f), and the HMGB1 level was analyzed by ELISA. All the data are representative of at least three independent experiments, and the data in (a), (c), (e), and (g) are presented as the mean ± SEM. *P < 0.05 versus DMSO vehicle and ^#^ *P* < 0.05 versus LPS alone.

It has been reported that both HMGB1 and PPAR*α* are constitutively expressed in cardiomyocytes [2]; therefore, we examined whether activation of PPAR*α* affects HMGB1 expression in primary cultures of neonatal rat cardiomyocytes. Fenofibrate induced a time-dependent reduction in HMGB1 expression at the mRNA level and protein level (Figures 1(a) and 1(b)), and this downregulation of HMGB1 was concentration dependent (Figures 1(c) and 1(d)). In addition, secretion of HMGB1 was also inhibited by fenofibrate (Figure 1(g)).

Considering that expression of HMGB1 may be increased in disease states, in addition to evaluating the influence of fenofibrate on HMGB1 expression at the basal level, we also investigated its effect on upregulation of HMGB1 by LPS. As shown in Figures 1(e) and 1(f), HMGB1 expression increased with LPS treatment. However, pretreatment with fenofibrate attenuated the LPS-induced upregulation of HMGB1. The HMGB1 level in the supernatant was also determined by ELISA and found to change in parallel with HMGB1 expression (Figure 1(g)).

### 3.2. Activation of PPAR by Fenofibrate Modulates the Localization of HMGB1

**Figure 2:**
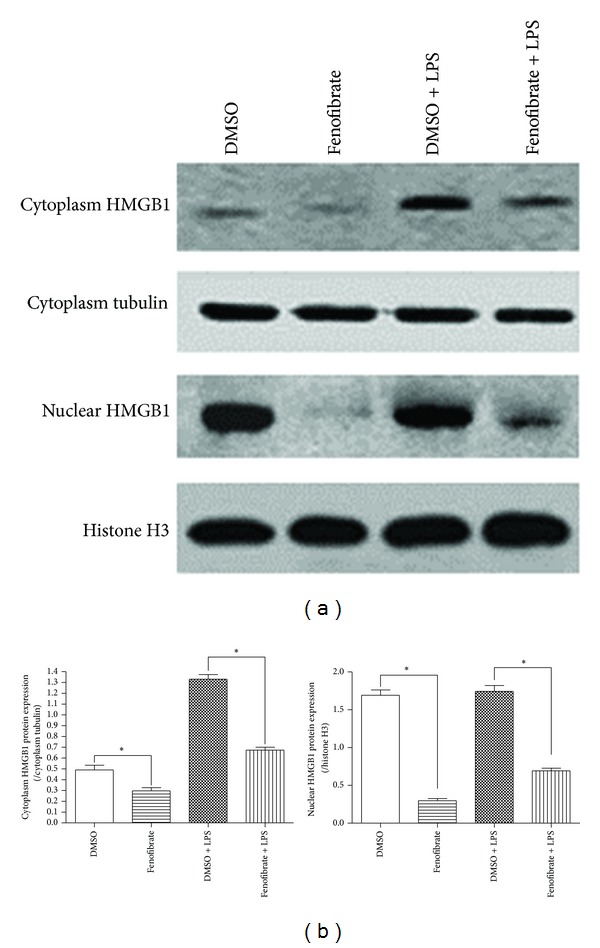
Fenofibrate modulates the localization of HMGB1 in cardiomyocytes. Cells were treated with 10 *μ*M fenofibrate or DMSO for 36 h and then with or without LPS stimulation (50 ng/mL) for 10 h. (a) Cells were analyzed by western blot to determine the effect of fenofibrate on the distribution of HMGB1 between the nucleus and cytoplasm. Histone H3 and cytoplasmic tubulin were used as loading controls for nuclear protein and cytoplasm protein, respectively. Data are representative of three independent experiments. (b) Densitometry results for western blot, *P < 0.05 as indicated comparisons; all data are expressed as means ± SEM, *n* = 3.

It is well known that HMGB1 is normally located in the nucleus as a chromatin-associated protein and that its function is dependent on its cellular location. However, it is still unknown whether fenofibrate modulates the nuclear-cytoplasmic translocation of HMGB1. Here, we detected the protein level of HMGB1 in the nucleus and cytoplasm of cardiomyocytes with or without fenofibrate treatment. As shown in Figure 2, HMGB1 was predominantly expressed in the nucleus of cardiomyocytes in the DMSO control group, and fenofibrate treatment decreased the protein level of HMGB1 in the nucleus compared to that for the DMSO control. However, LPS stimulation resulted in a dramatic increase in HMGB1 in both the nucleus and cytoplasm of cardiomyocytes, which was also inhibited by fenofibrate. Therefore, fenofibrate modulates HMGB1 expression and may also modulate its nuclear and cytoplasmic localization.

### 3.3. Expression and Location of HMGB1 in the Hearts of Mice with or without Cardiac Hypertrophy

**Figure 3:**
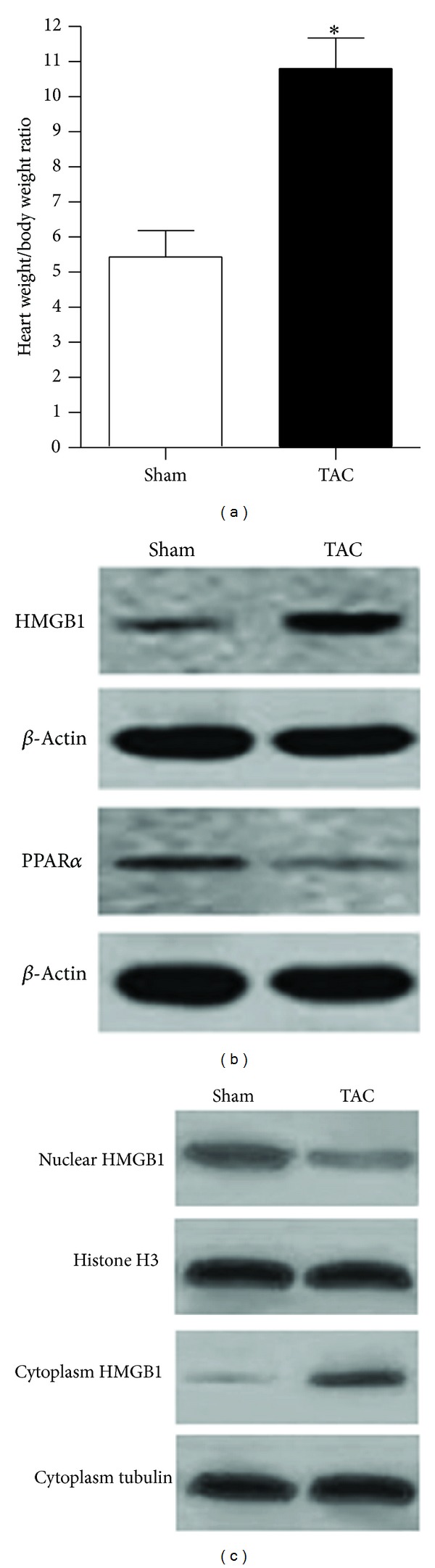
Decreased expression of PPAR*α* and increased expression of HMGB1 in hypertrophic hearts induced by TAC. (a) Heart weight : body weight in TAC and sham-operated mice at 4 weeks after the operation. (b) Decrease in PPAR*α* and increase in HMGB1 at the protein level in cardiomyocytes after the TAC operation; western blot was performed to analyze the expression of PPAR*α* and HMGB1, and *β*-actin was used as the loading control. (c) Translocation of HMGB1 from the nucleus to the cytoplasm after the TAC operation; histone H3 and cytoplasmic tubulin were used as loading controls for nuclear protein and cytoplasm protein, respectively. All of the data are representative of at least three independent experiments. The data in (a) are presented as the mean ± SEM for five mice from each group. *P < 0.05 versus sham-operated mice.

After demonstrating the effect of PPAR*α* on HMGB1 expression and localization in cardiomyocytes, we next investigated whether this effect could influence the progression of cardiac hypertrophy. A mouse model of cardiac hypertrophy induced by TAC was used to investigate the expression of both HMGB1 and PPAR*α* in the hearts of the model mice. Control mice were subjected to sham surgery. At 4 weeks after operation, both TAC mice and sham mice were sacrificed, and their hearts were rapidly excised for experiments. We found that the increase in the weight of the heart was significantly higher in TAC mice than in sham mice (Figure 3(a)), which means that the cardiac hypertrophy model was successfully constructed. We next examined the expression of HMGB1 and PPAR*α* in the hearts of the TAC group and sham group, and the results showed that PPAR*α* expression was higher in the sham group than in the TAC group. In contrast, HMGB1 expression was higher in TAC group (Figure 3(b)). In addition, we also determined the localization of HMGB1 in cardiomyocytes in both TAC and sham mice. Compared with the findings for sham-operated mice, the expression of nuclear HMGB1 in the TAC group was lower, whereas the expression of cytoplasmic HMGB1 was higher (Figure 3(c)), suggesting that the location of HMGB1 in cardiomyocytes may be associated with the progression of cardiac hypertrophy.

### 3.4. Fenofibrate Prevents the Development of Cardiac Hypertrophy Possibly by Modulating HMGB1 Localization

**Figure 4:**
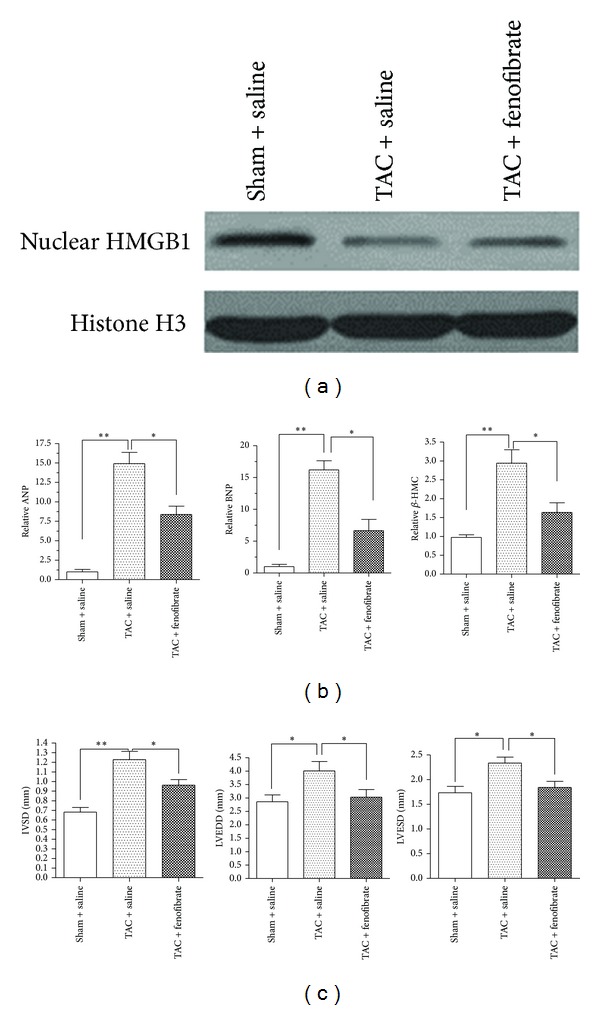
Fenofibrate suppresses the development of cardiac hypertrophy and increases the expression of nuclear HMGB1 in the heart. (a) Mice were treated with saline or the same dose of fenofibrate (50 mg/kg via gavage) after operation for 4 weeks; nuclear HMGB1 levels in the hearts of the mice were analyzed by western blot, and histone H3 was used as the loading control. (b) Mice were treated as described above, and ANP, BNP, and *β*-MHC gene expressions in mice were quantitatively analyzed by real-time PCR. All of the data are representative of at least three independent experiments, and the data in (b) are the mean ± SEM from five mice for each group. *P < 0.05 and **P < 0.01 as indicated in the comparisons. (c) Data showing echocardiographic measurements in mice treated as described above. IVS: interventricular wall thickness; LVEDD: left-ventricular end-diastolic dimension; LVESD: left-ventricular end-systolic dimension. Data are the mean ± SEM from three mice for each group. *P < 0.05 and **P < 0.01, as indicated in the comparisons.

The *in vitro* experiments clearly demonstrated that fenofibrate modulates HMGB1 expression and localization in cardiomyocytes. Additionally, nuclear HMGB1 in cardiomyocytes was also found to be upregulated in TAC mice. To determine whether fenofibrate also regulates HMGB1 *in vivo*, mice that underwent the TAC operation were randomly divided into two groups: one group received fenofibrate (50 mg/kg via gavage) daily for 4 weeks after the operation and the other group was administered saline. Sham-operated mice were used as normal controls and received saline rather than fenofibrate. Fenofibrate treatment increased the protein level of HMGB1 in the nucleus to a greater extent in the TAC group (Figure 4(a)).

We therefore subsequently determined whether fenofibrate-mediated promotion of HMGB1 translocation from the nucleus to the cytoplasm plays a role in the development of cardiac hypertrophy. Cardiac function at 4 weeks after the TAC or sham operation was evaluated by examining the mRNA expression of fetal cardiac genes. As shown in Figure 4(b), ANP, BNP, and *β*-MHC were significantly upregulated in the TAC group compared with the sham surgery group, and this upregulation was significantly attenuated when mice were treated with fenofibrate after the operation. Moreover, systolic dysfunction and left-ventricular dilatation after TAC were attenuated by fenofibrate treatment (Figure 4(c)). These findings demonstrate that fenofibrate inhibits cardiac hypertrophy and regulates HMGB1 translocation; therefore, we hypothesized that fenofibrate-mediated regulation of HMGB1 localization may also play a role in inhibiting the development of cardiac hypertrophy.

## 4. Discussion

In the present study, we found that activation of PPAR*α* by fenofibrate downregulates HMGB1 in cardiomyocytes and modulates their localization between the nucleus and cytoplasm. These effects of fenofibrate may also play a role in the development of cardiac hypertrophy. To our knowledge, this is the first study demonstrating that fenofibrate prevents cardiac hypertrophy development by inhibiting nuclear HMGB1 expression.

Thus far, several approaches have been used to target HMGB1, including the use of anti-HMGB1 antibodies, peptide antagonists, soluble receptors, and inhibitory molecules such as ethyl pyruvate and thrombomodulin [6, 20, 21]. In the present study, we found that activation of PPAR*α* by fenofibrate is also effective in inhibiting HMGB1 expression in cardiomyocytes at both basal and LPS-enhanced levels. To date, no study has addressed the role of PPAR*α* in regulating HMGB1 expression. However, a recent report demonstrated that PPAR*γ*-dependent inhibition of HMGB1 may play a role in inhibiting inflammatory reactions in postischemic injury [22]. Additionally, a previous investigation also showed that the PPAR*γ* agonist troglitazone inhibited HMGB1 expression in endothelial cells [19]. Because the physiological functions of PPAR*α* and PPAR*γ* are crossed in many diseases, fenofibrate is a logical candidate to block LPS-induced inflammation by inhibiting HMGB1 expression.

In addition to the finding that fenofibrate inhibited the expression of HMGB1, we also found that fenofibrate may modulate HMGB1 localization in the nucleus and cytoplasm. Previous studies have reported that posttranslational modification by acetylation of lysine residues in cultured cells modifies the binding of HMGB1 to DNA and the extranuclear localization of HMGB1 [23, 24]. Hyperacetylation of HMGB1 induced by lipopolysaccharide and hydrogen peroxide triggers its translocation into the cytoplasm [24–26]. A recent report also showed that, in cardiomyocytes, ET-1 and AngII promoted HMGB1 acetylation and subsequently suppressed the development of cardiac hypertrophy [2]. In this study, we demonstrated that fenofibrate maintained the level of nuclear HMGB1, which may affect the acetylation or phosphorylation of HMGB1. However, more evidence should be provided to determine the molecular mechanism underlying HMGB1 localization upon fenofibrate-induced PPAR*α* activation.

Several cell- and rodent-based studies have demonstrated PPAR*α*-dependent cardioprotection in the context of cardiac hypertrophy. Fenofibrate was found to inhibit endothelin-induced Akt and glycogen synthase kinase-3-*β* phosphorylation, thereby inhibiting the development of cardiomyocyte hypertrophy [27]. Nuclear translocation of the prohypertrophic transcription factor—nuclear factor of activated T cells (NFAT)—has been shown to be inhibited by fenofibrate [12, 27]. However, these results cannot fully explain the mechanism underlying the effect of fenofibrate in cardiac hypertrophy, and more evidence is required for this process. We showed that facilitation of PPAR*α* expression by fenofibrate was accompanied by reduction in HMGB1 expression. In addition, we found that nuclear HMGB1 levels decreased in the mice with cardiac hypertrophy and that fenofibrate treatment reversed this decrease and inhibited the development of this disease. Previous studies have shown that nuclear HMGB1 may play a role in preventing DNA damage in the brain and neurons [28, 29]. Recent experiments conducted by Funayama et al. [2] have demonstrated that maintaining the nuclear HMGB1 level may prevent DNA damage in cardiomyocytes, thus decreasing the severity of cardiac hypertrophy. Taken together, these results further support our hypothesis that activation of PPAR by fenofibrate might prevent the development of cardiac hypertrophy by modulating HMGB1 expression and localization.

There are several limitations to this study. In the present study, we showed that upregulation of nuclear HMGB1 by fenofibrate in cardiomyocytes and the heart prevents cardiac dysfunction after TAC operation; however, we did not find a direct link between increased nuclear HMGB1 levels and cardiac hypertrophy* in vivo*. To confirm such a relationship, it may be necessary to evaluate cardiac function in cardiac-specific HMGB1 knockout mice in a future study. However, we demonstrated that fenofibrate could prevent the development of cardiac hypertrophy by modulating HMGB1 expression and localization, which also provides a novel approach to the investigation of the pathogenesis of cardiac hypertrophy.

## Figures and Tables

**Table 1:** Primers for real-time PCR.

Genes	Primers
Rat HMGB1	Forward: 5′-AGCAATCTGAACTTCTGTCC-3′
	Reverse: 5′-GTTCTTGTGATAGCCTTCTC-3′

Rat PPAR*α*	Forward: 5′-CCCTCTCTCCAGCTTCCAGCCC-3′
	Reverse: 5′-CCACAAGCGTCTTCTCAGCCATG-3′

Mouse ANP	Forward: 5′-CAGCATGGGCTCCTTCTCCA-3′
	Reverse: 5′-TCCGCTCTGGGCTCCAATCCT-3′

Mouse BNP	Forward: 5′-CTGAAGGTGCTGCCCCAGATG-3′
	Reverse: 5′-GACGGATCCGATCCGGTC-3′

Mouse *β*-MHC	Forward: 5′-CTACAGGCCTGGGCTTACCT-3′
	Reverse: 5′-TCTCCTTCTCAGACTTCCGC-3′

Rat *β*-actin	Forward: 5′-TCATGAAGTGTGACGTTGCATCCGT-3′
	Reverse: 5′-CCTAGAAGCATTTGCGGTGCCGATG-3′

Mouse GAPDH	Forward: 5′-AACTTTGGCATTGTGGAAGG-3′
	Reverse: 5′-TGTGAGGGAGATGCTCAGTG-3′
